# Causal association between common rheumatic diseases and arrhythmia: a Mendelian randomization study

**DOI:** 10.3389/fcvm.2024.1419466

**Published:** 2024-10-01

**Authors:** Yuchen Zhang, Ling Tang, Ke Zhang, Xinai Meng, Tian Liu, Yanjia Chen, Xingfu Huang

**Affiliations:** ^1^ Department of Cardiology, Nanfang Hospital, Southern Medical University, Guangzhou, China; ^2^ Department of Neurology, Nanfang Hospital, Southern Medical University, Guangzhou, China; ^3^ Department of Anesthesiology, Nanfang Hospital, Southern Medical University, Guangzhou, China

**Keywords:** Mendelian randomization, arrhythmia, rheumatic diseases, single-nucleotide polymorphisms, RBBB

## Abstract

**Background:**

Observational studies have suggested a link between rheumatic diseases and arrhythmias. However, these studies have been limited by confounding factors and reverse causality, leaving the causal relationship between rheumatic diseases and arrhythmias uncertain. This study addresses this inquiry using genetic evidence.

**Methods:**

Selected single nucleotide polymorphisms (SNPs) from genome-wide association study (GWAS) data were employed as instrumental variables. Inverse variance weighting (IVW), MR-Egger regression, and the weighted median method were utilized in the two-sample Mendelian randomization analysis. Horizontal pleiotropy was identified and rectified through the MR-PRESSO test and MR-Egger regression. The stability and reliability of the Mendelian randomization results were appraised using the remain-one method, Cochran *Q*-test, and funnel plot. Odds ratios (OR) were utilized to assess the causal relationship between six rheumatic diseases and five types of arrhythmias.

**Results:**

The Inverse Variance Weighted (IVW) method indicated a significant association between rheumatoid arthritis (RA) and an elevated risk of right bundle branch block (RBBB) (OR: 1.10, 95% CI: 1.02–1.18, *p* = 0.009). Additionally, gout was significantly correlated with an augmented risk of RBBB (OR: 1.28, 95% CI: 1.09–1.51, *p* = 0.003). Conversely, dermatomyositis (DM) exhibited a negative association with the risk of atrioventricular block (AVB) (OR: 0.94, 95% CI: 0.90–0.99, *p* = 0.020). No significant associations were observed between other rheumatic diseases and arrhythmias.

**Conclusion:**

A two-sample Mendelian Randomization (MR) study provides data indicating that in European populations, a genetically predicted gout or rheumatoid arthritis (RA) may increase the incidence of right bundle branch block (RBBB). To clarify and investigate the processes behind these causal links, more research is necessary. Because racial genetic variability exists, care should be used when interpreting our findings.

## 1 Introduction

Arrhythmias can be classified as tachyarrhythmias or chronic arrhythmias based on heart rate. Bradycardia and atrioventricular block are the primary characteristics of chronic arrhythmias, whereas tachycardias are primarily characterized by tachycardia, supraventricular tachycardia, ventricular fibrillation, atrial fibrillation, etc. (1). Some arrhythmias are asymptomatic and transitory, while others are persistent and carry a significant burden on the healthcare system due to the potential for hemodynamic instability, thromboembolic events, and even sudden cardiac death (2, 3). Despite this, evidence for effective prevention and treatment of arrhythmias has been limited until now (4). In conclusion, it is imperative to investigate unidentified risk factors for arrhythmias, facilitating early detection and prompt treatment to mitigate the harm associated with these irregular heartbeats.

Over 100 disorders fall under the category of rheumatic diseases, which are a group of autoimmune and/or inflammatory conditions that can harm organs, joints, muscles, and bones. In the US, rheumatism ranks higher than heart disease, diabetes, and cancer as the leading cause of disability among people (5, 6). Rheumatic diseases are conditions that lead to tissue damage within the body due to immune dysfunction, frequently affecting multiple organs and systems (7). Among these, the heart stands out as a frequent target organ for autoimmune diseases. The comprehensive structure of the heart may become implicated, giving rise to disturbances in microcirculation, myocardial fibrosis, and compromised valve function (8–10). However, it is noteworthy that arrhythmias, despite being a prevalent cardiac condition, have been relatively overlooked within the realm of rheumatology.

Several earlier studies have demonstrated that risk factors for arrhythmia, such as smoking, diabetes, hypertension, and dyslipidemia, increase the likelihood of developing arrhythmia (11). Addressing these risk factors can help doctors implement novel therapies, reduce the financial burden on patients, and decrease the incidence of arrhythmias (12). Some research suggests a connection between arrhythmias and rheumatic disorders, although the exact mechanism—possibly involving inflammation and autoimmunity—remains unclear (13–15). Li et al. introduced the concept of the “gut-immune-heart” axis, linking gut flora to atrial fibrillation (16). Several omics studies have demonstrated both direct and indirect causal links between gut microbiota and atrial fibrillation. Over time, numerous studies have explored the causal relationship between rheumatic disorders and cardiovascular diseases (17). A meta-analysis revealed that patients with rheumatoid arthritis (RA) may be more susceptible to atrial fibrillation (18). Previous case-control studies have shown that gout sufferers have a higher incidence of arrhythmia (19). Similarly, hyperuricemia in AF patients has been independently associated with an increased risk of hospitalization for heart failure and all-cause mortality (20). In summary, there is a potential correlation between rheumatic diseases and arrhythmias, and this paper aims to determine whether a causal relationship exists between the two through rigorous scientific methods.

Mendelian randomization (MR) is a proven method in epidemiology for drawing causal inferences (21). It uses genetic variants from genome-wide association studies (GWAS) as instrumental variables (IVs) to evaluate how specific exposures affect a phenotype (22). Mendelian rules of inheritance govern genetic variation in MR, which is analogous to randomization in randomized controlled trials (RCTs) (23). Genetic variants associated with exposure factors serve as IVs, facilitating the inference of causal effects on study outcomes. Since genetic variation is intrinsic and unaffected by typical confounders, MR approaches are useful for concluding causality in observational investigations. These techniques are frequently employed to confirm causal links, and discoveries in genomics improve MR analysis by identifying more GWAS disease-associated genetic variants (24).

Only ankylosing spondylitis (AS), rheumatoid arthritis (RA), systemic lupus erythematosus (SLE), Sjogren's syndrome (SS), dermatomyositis (DM), and gout are currently included in the most comprehensive GWAS database in rheumatology; as a result, the inclusion of these six diseases in this study represents common rheumatic diseases. We looked into how they could affect the following five main arrhythmias: atrial fibrillation (AF), atrioventricular block (AVB), right bundle branch block (RBBB), left bundle branch block (LBBB), and paroxysmal tachycardia (PT). Using aggregated level data from many major associations, we evaluated the causal link between arrhythmia and rheumatism for the first time in this two-sample MR (TSMR) survey.

## 2 Material and methods

### 2.1 Study design

**Figure 1:**
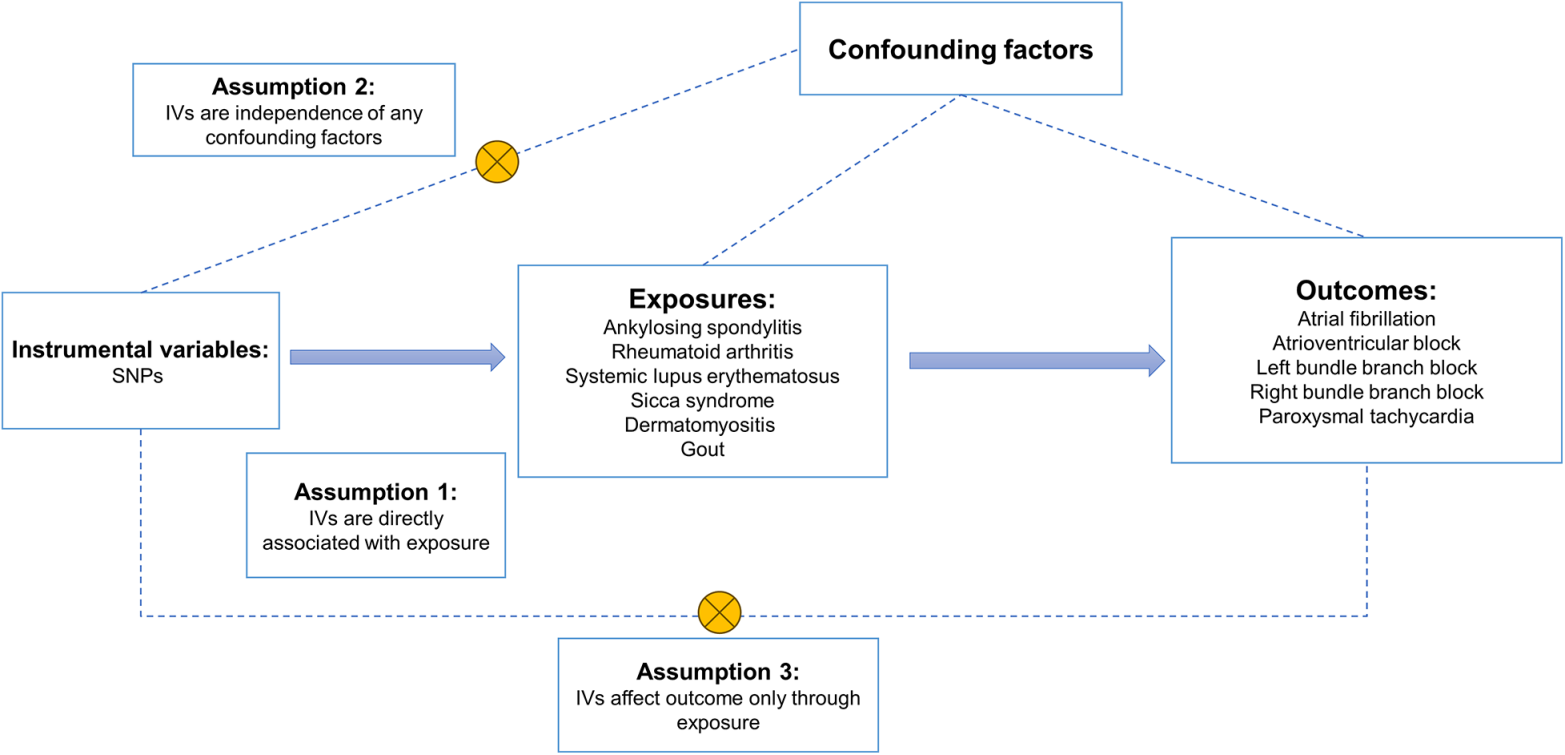
An overview of the study design. SNPs, single nucleotide polymorphisms; IVs, instrumental variables.

A two-sample MR analysis was conducted in accordance with the criteria established by the Strengthening Observational Studies in Epidemiology Using Mendelian Randomization Report (STROBEMR) to examine the association between the risk of five cardiac arrhythmias and six common rheumatic diseases (25). Since the previously collected summary-level data were reanalyzed, no further ethical approval was needed. R (version 4.3.1) and TwoSampleMR (version 0.5.8) were used for the MR analysis. For two-sample MR to be realized, three prerequisites had to be met. First, as instrumental variables (IVs), we chose genetic variations that are highly correlated with exposure (Rheumatic diseases). Second, there is no correlation between instrumental variables and either known or unknown confounders. Thirdly, there was no alternative way that the instrumental factors altered the outcome (arrhythmias) except through exposure. An overview of the research design is given in Figure 1.

### 2.2 Data source

For AS, RA, SLE, and AF, data were taken from the publicly accessible IEU Open GWAS database (https://gwas.mrcieu.ac.uk/). AVB, LBBB, RBBB, PT, DM, SS, and Gout data were sourced from the FinnGen9 database (http://www.finngen.fi). The sample sizes for each rheumatic illness were as follows: RA (14,361 cases and 43,923 controls), SLE (5,201 cases and 9,066 controls), DM (143 cases and 365,533 controls), gout (8,489 cases and 3,240,862), SS (2,495 cases and 365,533 controls), and AS (9,069 cases and 1,550 controls). The following were the sample sizes for arrhythmia: PT (9,604 cases and 191,924 controls), LBBB (1,918 cases and 286,109 controls), AVB (5,536 cases and 286,109 controls), and AF (60,620 cases and 970,216 controls). Further details on data sources are included in Supplementary Table S3. The analysis only included people of European ancestry, and for arrhythmia and rheumatic disease, there was less than 20% overlap between the cohorts.

### 2.3 Selection of IVs

We initially evaluated instrumental variables for six rheumatic diseases and five arrhythmias in order to investigate the causal link between them. Based on the following standards, single nucleotide polymorphisms (SNPs) were chosen as instrumental variables:(i) The genome-wide *p*-value of the SNP was <5 × 10^−8^ and thus the SNP was strongly associated with exposure, and because there were no genome-wide significant SNPs in DM, we used a less stringent threshold of 5 × 10^−6^ to obtain more SNPs for these phenotypes (26); (ii) SNPs with linkage disequilibrium (LD) r^2^ should be <0.001 and associated with index variants <10,000 KB. A low chance of weak IV bias is indicated by an *F*-value of less than 10. The following is the formula: F = (β_exposure_/SE _exposure_)^2^ denotes that the exposure's effect value and standard error were represented by β_exposure_ and SE_exposure_, respectively (23, 27). (iii) We also examined selected SNPs in Phenoscanner, a frequently used database of human genotype-phenotype correlations (www.phenoscanner.medschl.cam.ac.uk), to see if these SNPs were associated with pertinent confounders (28). (iv) Palindromic SNPs with intermediate allele frequencies were not included in the MR analysis. (v) In order to determine if the R2 of exposure exceeded the R2 of outcome, we lastly ran a Steger's test for each SNP. SNPs that tested “FALSE” were not included (29). See Supplementary Table S1 for details.

### 2.4 Statistical analysis

MR-Egger, weighted median (WM), and inverse variance weighting (IVW) are the three main mixed-methods (MR) approaches for identifying causal effects. IVW was the principal MR method employed in this investigation to evaluate the causative connection between arrhythmias and rheumatic diseases (30). The IVW technique, a meta-analysis that combines the Wald ratios for each SNP, is the basis for the concept that IV can only affect the outcome through specific exposures. When there is no horizontal pleiotropy in any SNP, the IVW technique offers a fair assessment of causation. In addition to MR analysis, the WM methodology and the MR-Egger method are utilized to evaluate bias resulting from faulty IVs and horizontally pleiotropic IVs. Using the MR-Egger technique might result in inaccurate estimations due to potential genetic variation. When over half of the IVs are horizontally pleiotropic, the WM technique has a minor bias but inferior precision. In addition to the MR analysis, we employed the WM approach to evaluate bias resulting from invalid IVs and horizontally pleiotropic IVs (31). We estimated causal estimates as odds ratios (ORs) per unit of standard deviation (SD) for continuous characteristics and ORs per unit of log odds ratio outcomes for categorical traits. The MR analysis data must undergo many sensitivity tests in order to assess potential heterogeneity and horizontal pleiotropy. We utilized the Cochran' *Q*-test to assess the instrumental factors' heterogeneity. The fixed-effects IVW approach was regarded as the primary tactic if the *p*-value was >0.05, indicating no heterogeneity; if not, a random-effects model was created (32). The identification of horizontal multinomial effects was done using the *p*-values for the intercept in MR-Egger. When the *p*-value was greater than 0.05, horizontal pleiotropy was not seen. To investigate the impact of removing one of the chosen individual SNPs on the outcome, we used leave-one-out analyses (33).

## 3 Results

### 3.1 Instrumental variables

In order to predict the genetic makeup of rheumatic diseases, a total of 179 SNPs were analyzed. These included 26 SNPs for AS, 90 SNPs for RA, 40 SNPs for SLE, 7 SNPs for SS, 3 SNPs for DM, and 13 SNPs for gout. Supplementary Table S1 provides comprehensive details regarding these SNPs linked to rheumatic diseases. All of the SNPs in this analysis had F-statistics more than 10, indicating a low probability of weak IV bias. Furthermore, the *p*-value threshold of 5 × 10^−6^ indicated a poor relationship between the SNPs and the outcomes, except for the DM SNP, for which the threshold was greater than 5 × 10^−8^.

### 3.2 Causal effect from rheumatic diseases on arrhythmias

**Figure 2:**
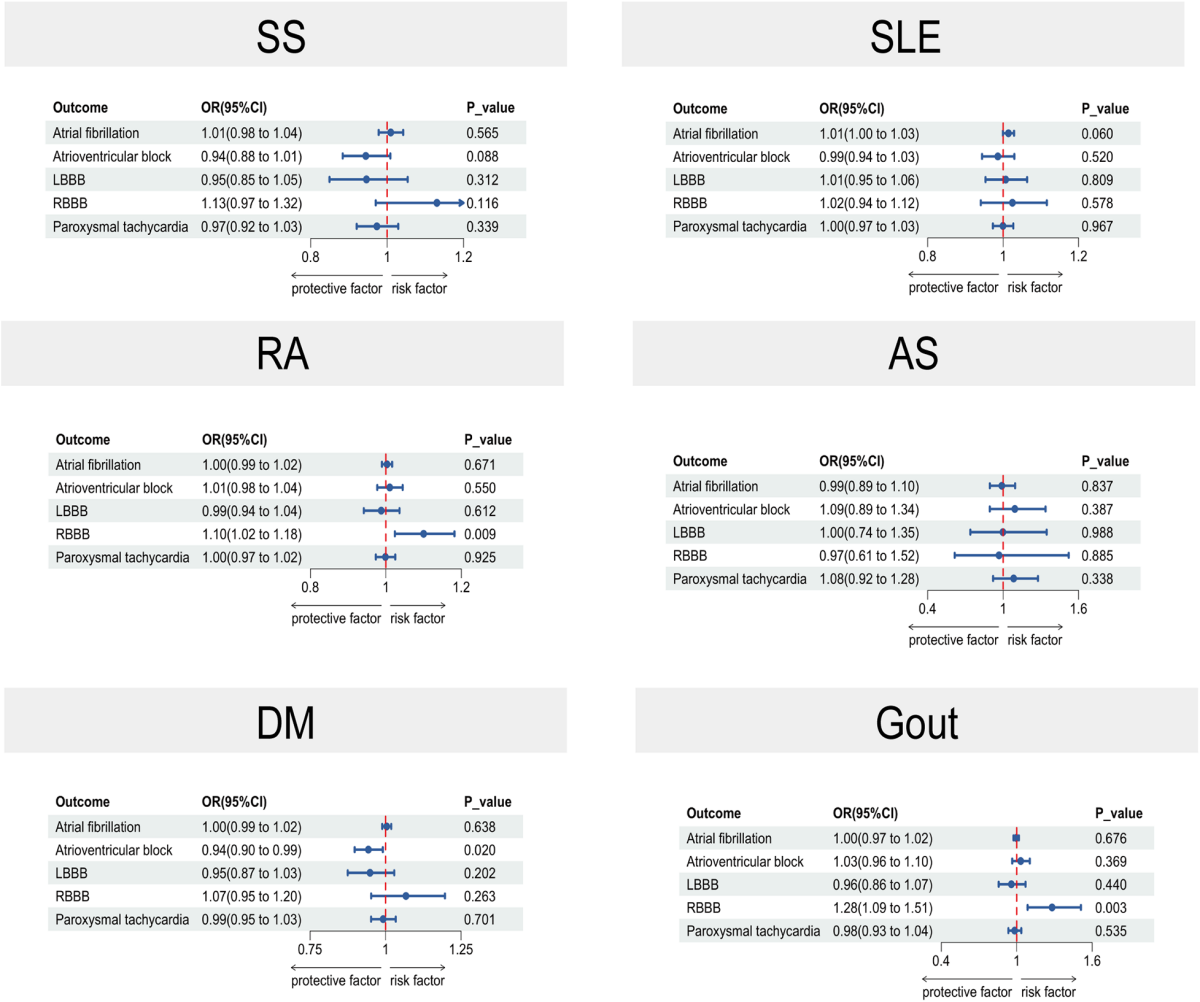
A forest plot in two-sample MR illustrating the causal estimates between arrhythmias and rheumatic illnesses. The horizontal solid line in the forest plot shows the 95% confidence interval (CI) for the associated OR value, whereas the dashed vertical line indicates the ineffective line (OR = 1). SLE, systemic lupus erythematosus; DM, dermatomyositis; SS, Sicca syndrome; RA, rheumatoid arthritis; AS, ankylosing spondylitis; CI, confidence interval.

Figure 2 lists the two-sample MR data for five cardiac arrhythmias and six rheumatic diseases. The risk of DM was found to have a negative correlation with AVB based on causal estimations derived from the IVW technique (OR: 0.94, 95% CI: 0.90–0.99, *p* = 0.020), genetically predicted RA was associated with RBBB (OR: 1.10, 95% CI: 1.02–1.18, *p* = 0.009), and Gout was associated with RBBB (OR: 1.28, 95% CI: 1.09–1.51, *p* = 0.003). Standard IVW analyses for SS, SLE, RA, AS, DM, and Gout did not show any significant effects on AF susceptibility (SS: OR = 1.01; 95% CI, 0.98–1.04; *p* = 0.565; SLE: OR = 1.01; 95% CI, 1.00–1.03; *p* = 0.060; RA: OR = 1.00; 95% CI, 0.99–1.02; *p* = 0.671; AS: OR = 0.99; 95% CI, 0.89–1.10; *p* = 0.837; DM: OR = 1.00; 95% CI, 0.99–1.02; *p* = 0.638; gout: OR = 1.00; 95% CI, 0.97–1.02; *p* = 0.676). Other rheumatic diseases were not significantly associated with arrhythmia.

### 3.3 Sensitivity analyses of MR

Supplementary Table S2 shows Cochran's Q statistic, the results of the MR-Egger intercept test, and the MR-PRESSO. All Cochran's Q-derived *p*-values were more than 0.05, except AS to PT, RA to AF, RA to AVB, SLE to AF, and SLE to AVB. All *p*-values for the MR-Egger intercept test were greater than 0.05, which suggests that horizontal pleiotropy is not present. All *p*-values in the MR-PRESSO test were greater than 0.05, except DM, as the sample size of SNPs was insufficient to conduct the test. By removing each SNP individually during a leave-one-out test, we were able to demonstrate the stability of the MR estimations (Supplementary Figure S1).

## 4 Discussion

Research shows that rheumatoid arthritis and gout are associated with a higher risk of right bundle branch block. Moreover, our results suggest a possible causative connection between AV block and dermatomyositis. No genetic causal relationship between any other rheumatic disease and other cardiac arrhythmias was found in this two-sample MR study.

Multiple organs and systems are impacted by the vast range of rheumatic illnesses. Indeed, a common extra-articular symptom of many rheumatic illnesses, including gout and rheumatoid arthritis (RA), is cardiac involvement (34). For instance, Villecco et al. reported a right bundle branch block in 35% of 60 patients diagnosed with rheumatoid arthritis (RA). These patients exhibited significantly higher frequencies of antibodies to cardiac conduction tissue compared to those without conduction abnormalities (76% vs. 21%) (35). Additionally, a cross-sectional study demonstrated a heightened prevalence of conduction abnormalities among patients with rheumatic diseases compared to the general population. The likelihood of conduction abnormalities rises with advancing age (36). Our Mendelian Randomization (MR) study identified genetic causality between rheumatoid arthritis (RA) and right bundle branch block (RBBB) and between gout and RBBB. The co-occurrence of RBBB in patients with RA or gout may be associated with various factors, including autoimmune and inflammatory elements. The specific pathogenesis of RBBB in patients with RA and gout remains unknown due to the limited number of relevant studies. However, potential mechanisms include: (i) interaction resulting in RBBB between immune cells, fibroblasts, and/or cardiomyocytes, (ii) direct involvement of immune cells in the electrical remodeling of leukocytes resulting in RBBB (13, 37, 38), and (iii) In recent animal research, Dai et al. found that in RA rats, RA can cause atrial fibrillation (AF) by upregulating the production of inflammatory markers such TNF-α and IL-6 and increasing the number of cardiac fibroblasts. These inflammatory variables may be the cause of RBBB by supporting pathological mechanisms including autophagy and autonomic remodeling (39).

Prior investigations have highlighted the correlation between dermatomyositis and arrhythmia. Specifically, a retrospective study in the United States revealed a notable association between arrhythmias and substantial mortality in young and middle-aged patients with dermatomyositis compared to matched controls (40). However, the current literature lacks data regarding the prevalence of right bundle branch block (RBBB) in dermatomyositis (DM). This gap underscores the need for heightened attention to RBBB in the DM group. In our Mendelian Randomization (MR) study, we identified a genetic causal link between dermatomyositis (DM) and atrioventricular block (AVB). Nevertheless, despite leveraging Genome-Wide Association Study (GWAS) data from the latest Finnish database for dermatomyositis, the number of Single Nucleotide Polymorphisms (SNPs) obtained after addressing linkage imbalance remains limited to 3. This constraint raises concerns about the robustness of the causal inference we derived.

Our investigation identified no significant correlation between AF and SLE, AS, gout, DM, RA, or SS, in agreement with findings from several other studies. A Mendelian randomization study utilizing different data sources similarly demonstrated that genetically predicted SLE is not causally associated with AF risk (41). Additionally, previous studies have not established a significant correlation between AS and AF (42–44). A separate study on gout found that the association with AF was significant only in the Chinese population, whereas no such association was observed in the Japanese population (45). In contrast to our results, a retrospective cohort study reported a higher incidence of AF in individuals with dermatomyositis compared to matched controls (46). Moreover, another Mendelian randomization study based on East Asian populations identified a causal link between RA and AF in East Asians (47). As our study focused on European populations, these discrepancies may be attributed to ethnic differences. Notably, no research has yet been published exploring the relationship between AF and Sjogren's syndrome. Further studies are required to investigate the potential association between rheumatic immune disorders and AF.

Numerous noteworthy benefits of this study exist. Initially, this is the first investigation of the cause-and-effect link between six prevalent rheumatic illnesses and arrhythmias. Furthermore, the reliability of traditional observational studies for drawing causal conclusions is reduced due to issues with reverse causality and confounding variables. The study's Mendelian Randomization (MR) Design successfully resolves the majority of confounding variables while allaying worries about reverse causation. In addition, MR Research has benefits over traditional research methods in terms of convenience, cost-effectiveness, and labor intensity reduction.

It is important to acknowledge the limitations of this study. First, despite using MR-PRESSO testing to identify and eliminate aberrant single nucleotide polymorphisms (SNPs), there remains a possibility that heterogeneity could affect the research outcomes. Second, our findings may not be broadly applicable to other populations, as the GWAS dataset includes only individuals of European descent. Consequently, additional validation with larger and more diverse databases is necessary. Third, although we identified a causal relationship between rheumatoid arthritis (RA) and right bundle branch block (RBBB), gout and RBBB, and dermatomyositis (DM) and atrioventricular block (AVB), the lack of corresponding basic research limits our ability to determine whether common risk factors, molecular signaling pathways, or immune responses exist among RA, gout, DM, and arrhythmias. As a result, we cannot fully explain the mechanisms underlying these associations. Fourth, the instrumental variables currently used do not fully account for the influence of external regulatory factors, as the incidence and severity of DM, gout, and RA are influenced by a variety of independent host metabolic factors and susceptibilities. Future studies may require multiple side-by-side Mendelian randomization analyses to provide a more comprehensive and accurate understanding. Finally, the available GWAS data did not differentiate between disease severity (mild, moderate, or chronic) or distinguish between newly diagnosed and long-standing conditions. These various aspects of the rheumatic diseases under study may significantly influence the host's vulnerability to arrhythmias, and further exploration of these issues will be possible as more comprehensive data become available.

## 5 Conclusion

This is the first MR Study to examine the connection between five arrhythmias and six major rheumatic disorders (DM, gout, AS, RA, SLE, SS, and SS). We discovered a causative association between DM and AVB as well as a causal relationship between RA or Gout and RBBB (i.e., RA or Gout are risk factors for RBBB). Our findings shed light on the part that autoimmune diseases play in the etiology of arrhythmias.

## Data Availability

The original contributions presented in the study are included in the article/Supplementary Material, further inquiries can be directed to the corresponding author.
