# Supplementary material for: Causal association between common rheumatic diseases and arrhythmia: a Mendelian randomization study
Source: Front Cardiovasc Med. 2024 Oct 1;11:1419466. doi: 10.3389/fcvm.2024.1419466 (PMC11473426; doi:10.3389/fcvm.2024.1419466)
Supplement: Supplementary file 1 [file Datasheet1.pdf]

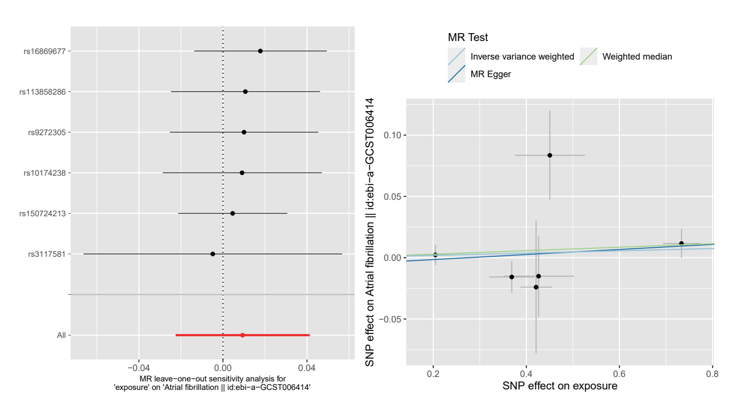

SS to AF

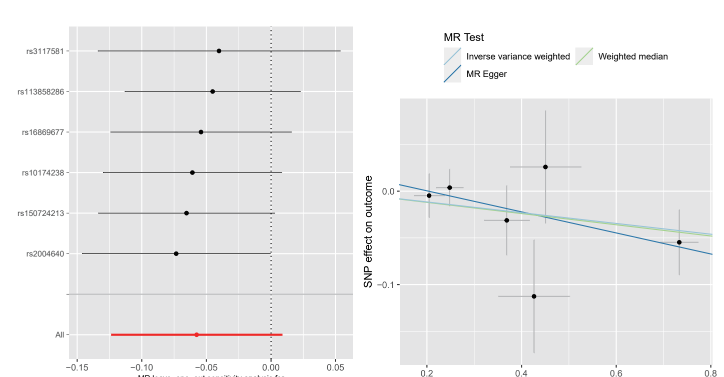

SS to AVBLock

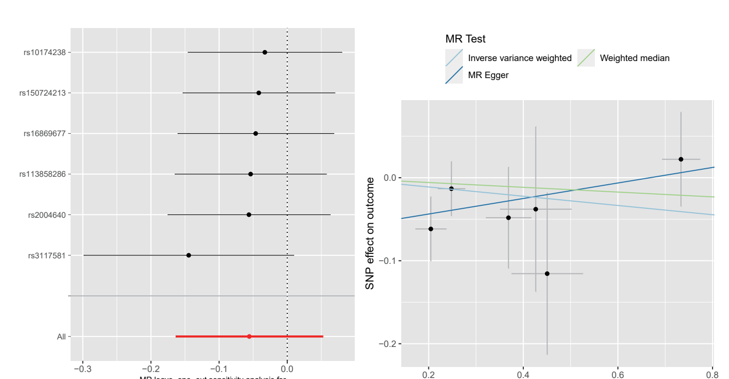

SS to LBBB

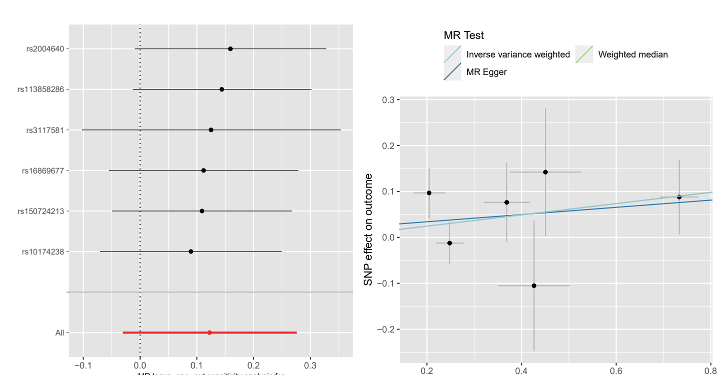

SS to RBBB

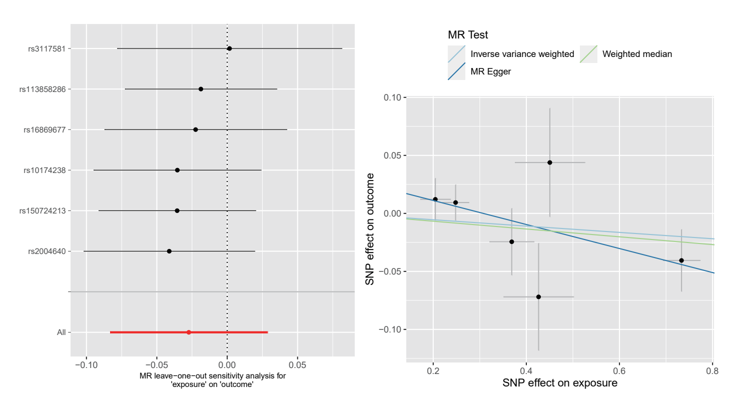

SS to PT

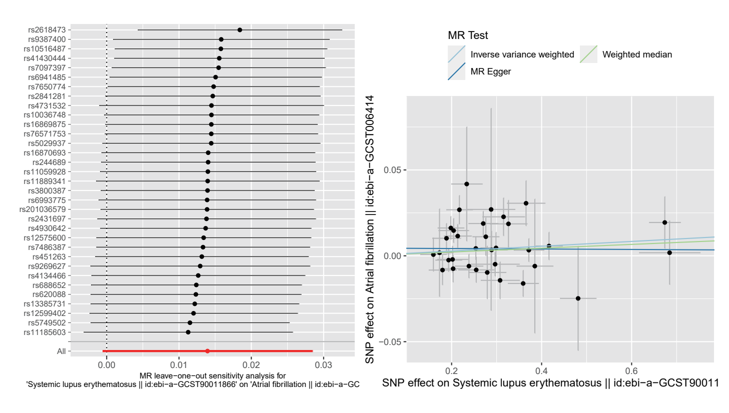

SLE to AF

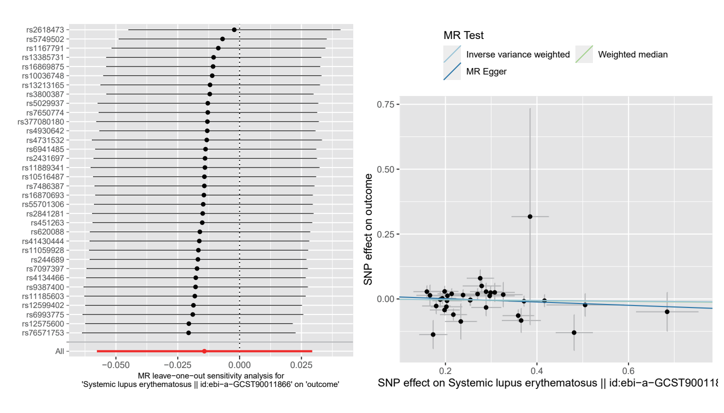

SLE to AVBlock

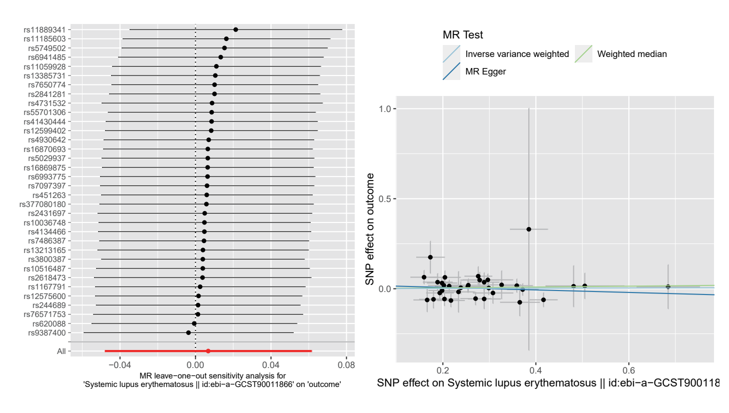

SLE to LBBB

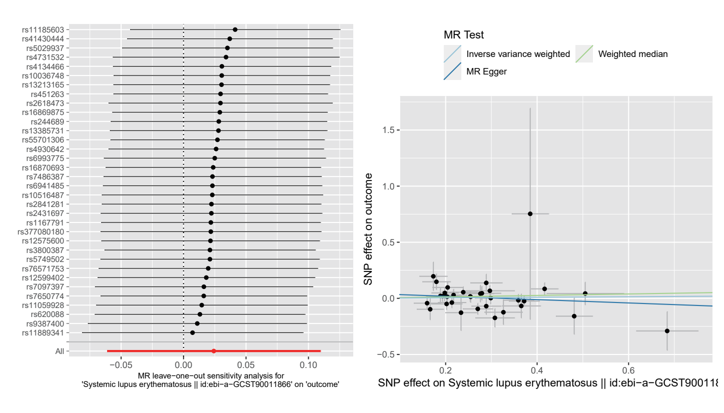

SLE to RBBB

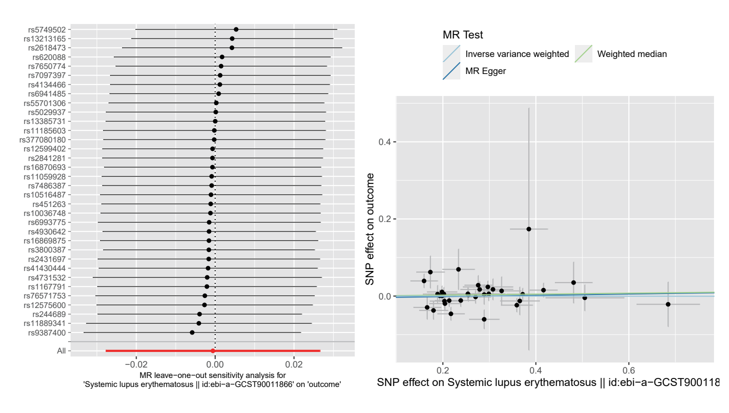

SLE to PT

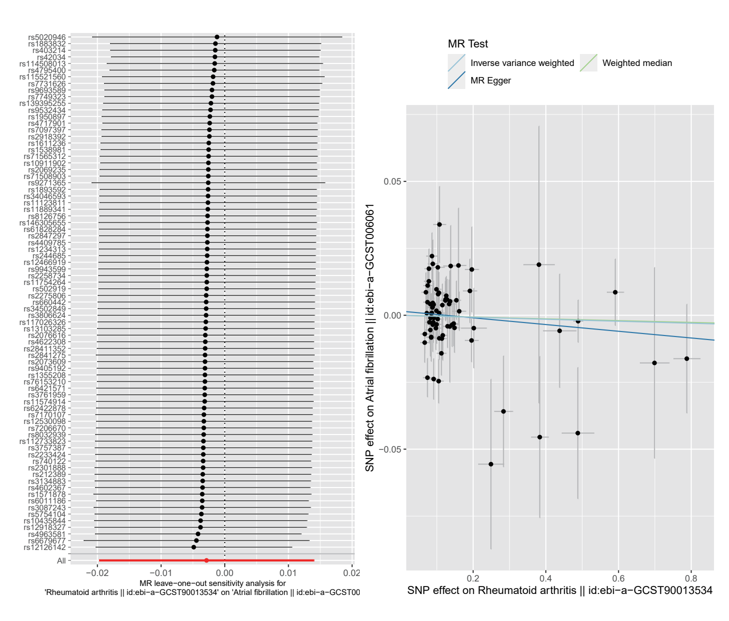

RA to AF

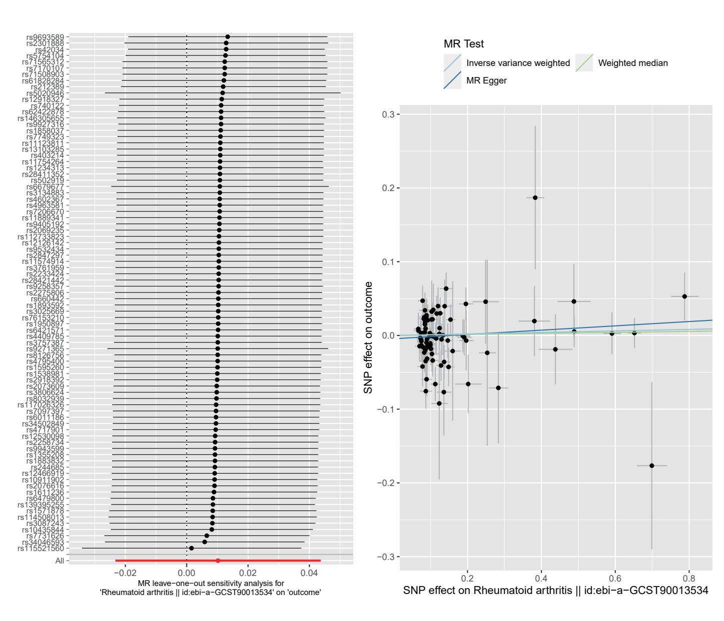

RA to AVBlock

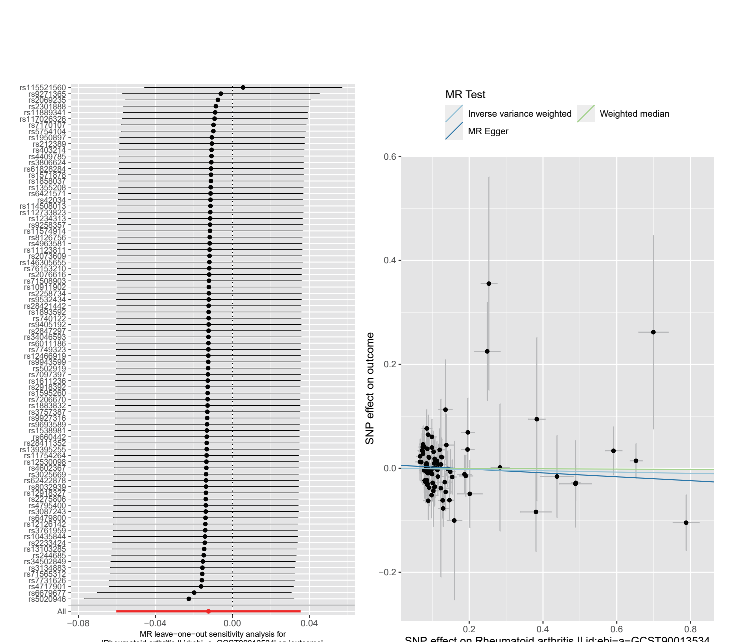

RA to LBBB

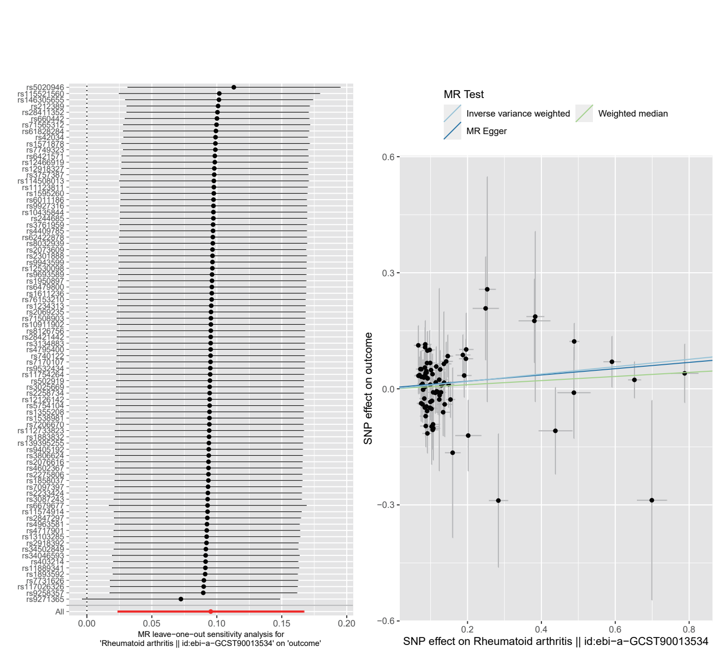

RA to RBBB

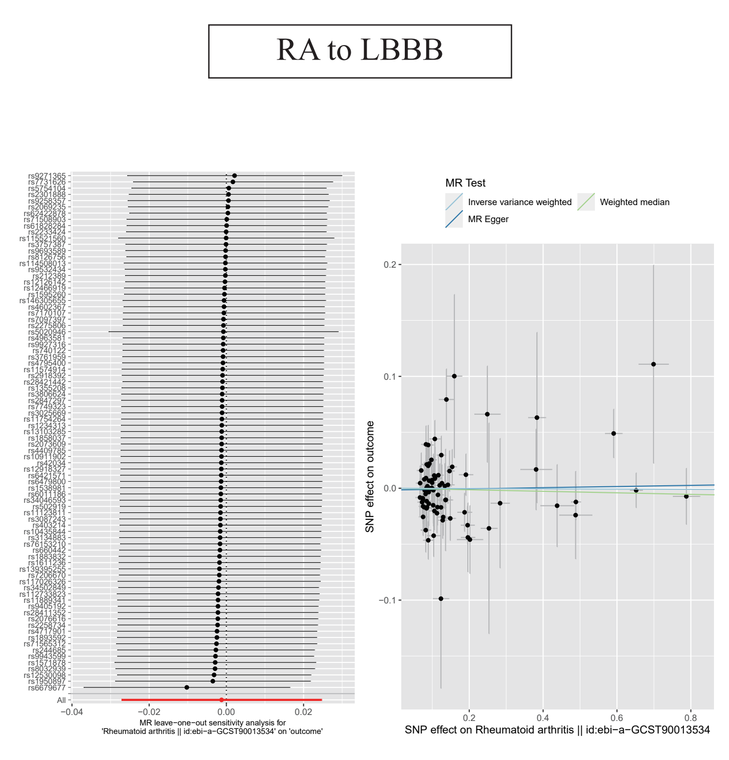

RA to PT

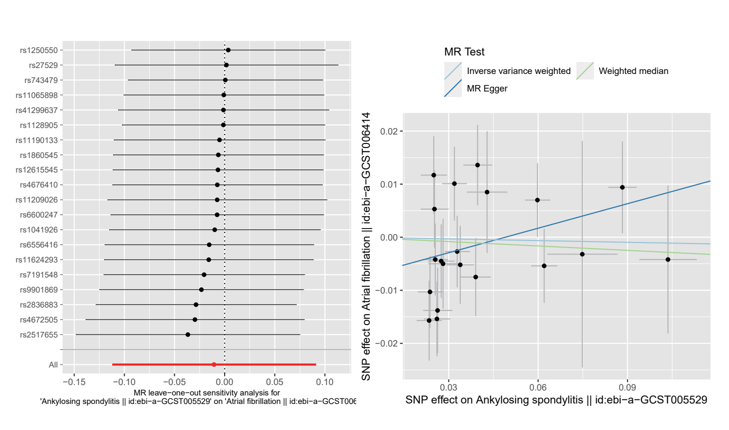

AS to AF

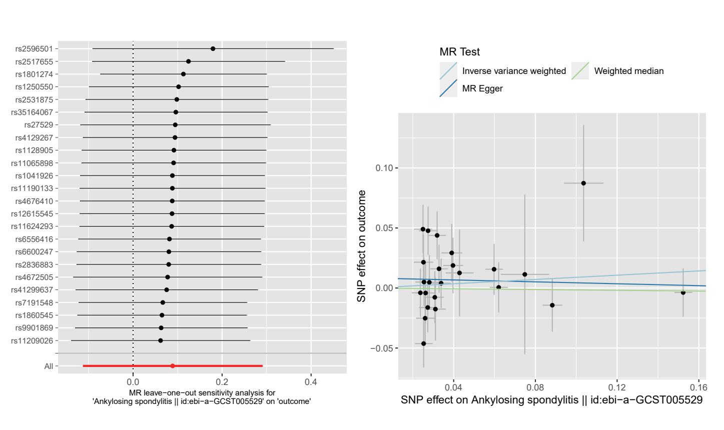

AS to AVBlock

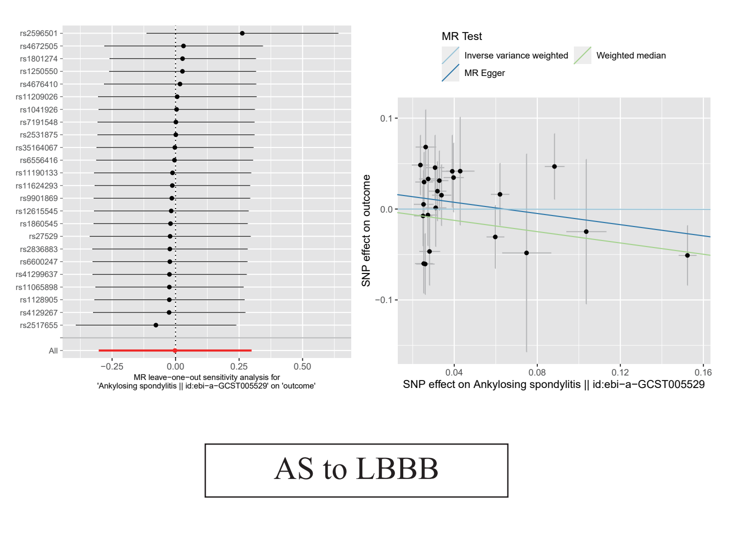

AS to LBBB

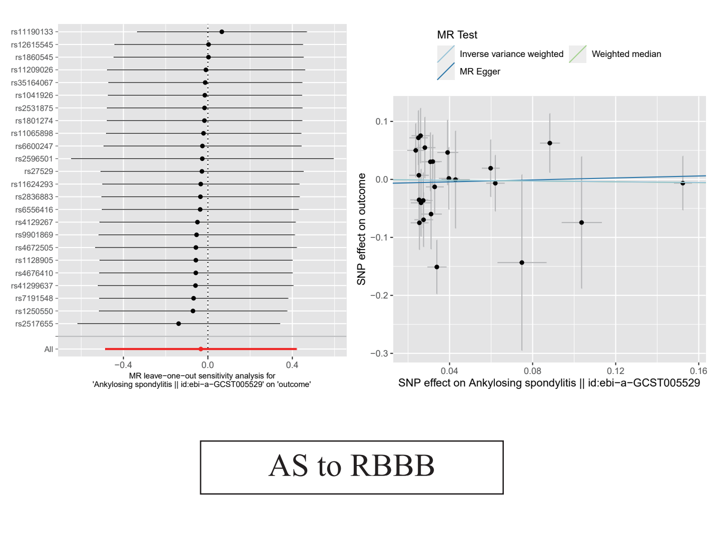

AS to RBBB

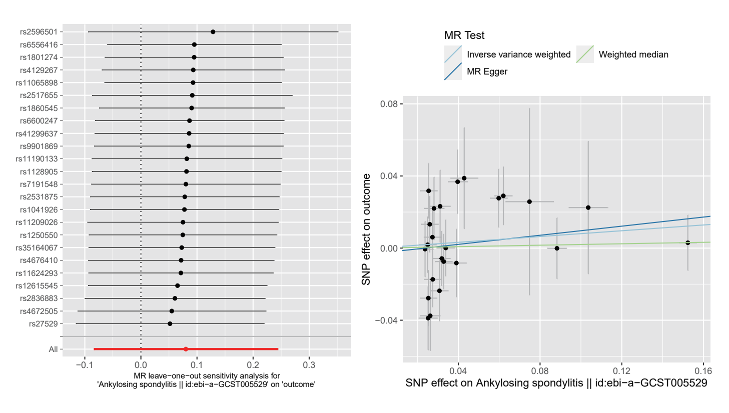

AS to PT

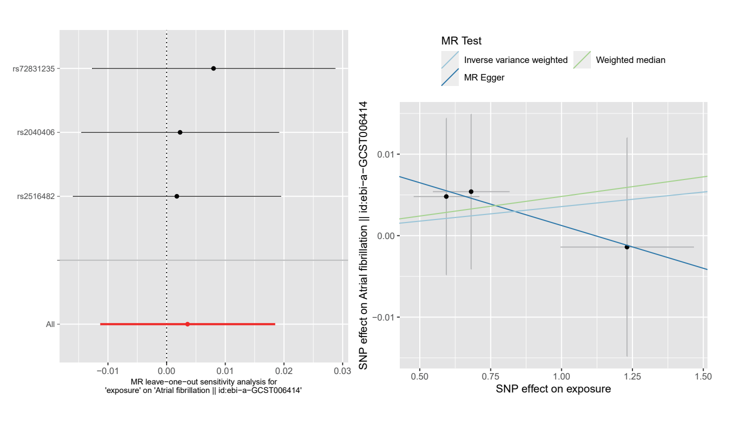

DM to AF

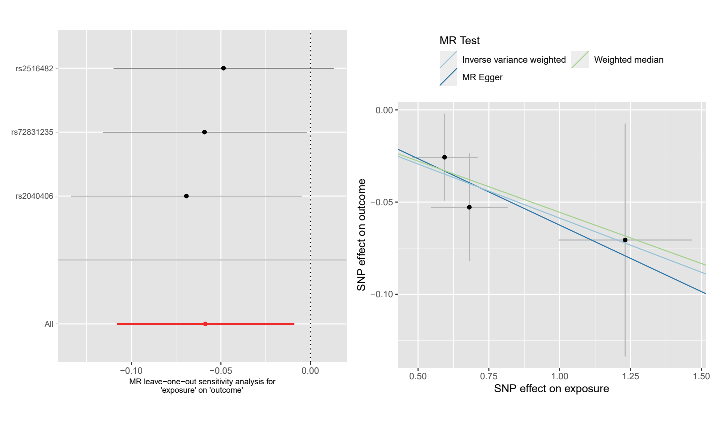

DM to AVBlock

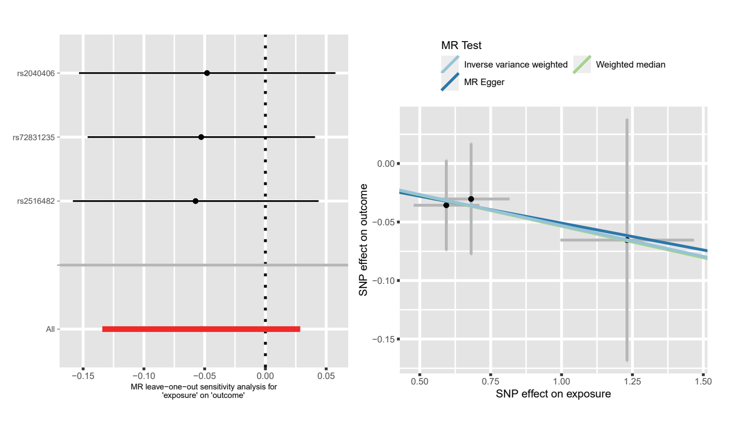

DM to LBBB

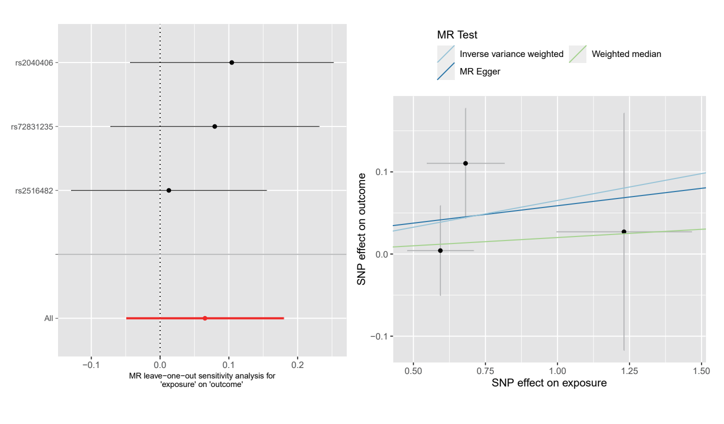

DM to RBBB

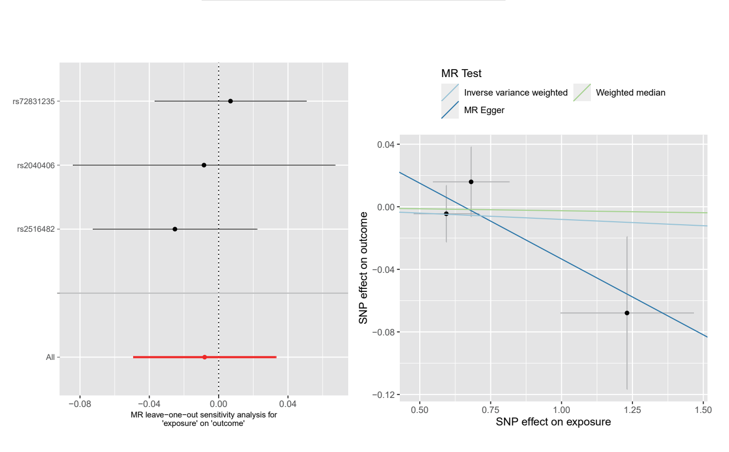

DM to PT

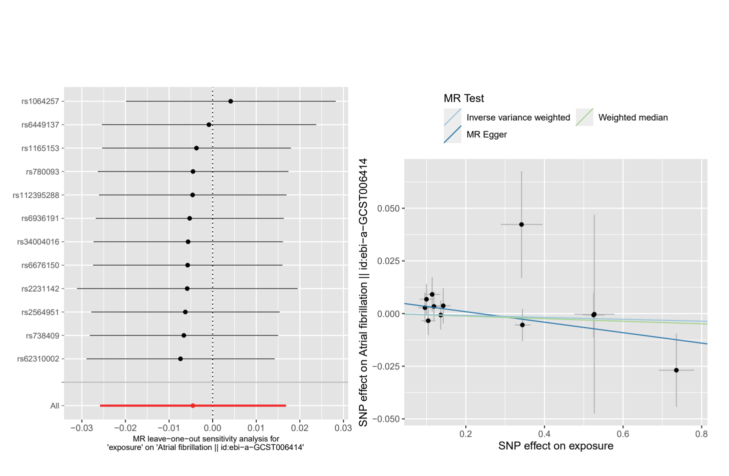

Gout to AF

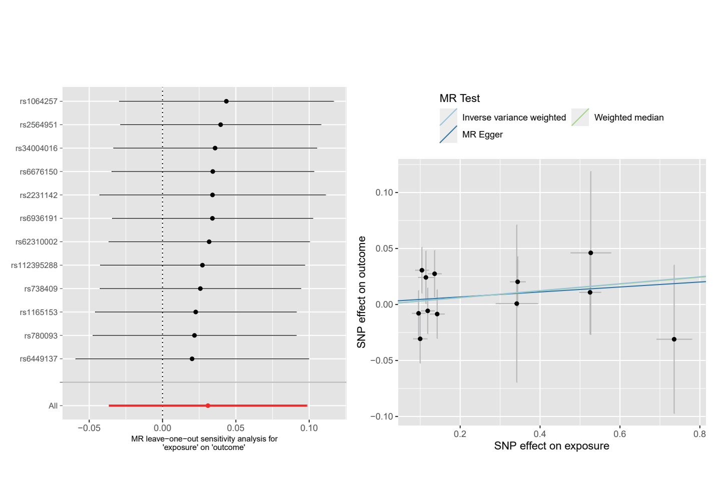

Gout to AVBlock

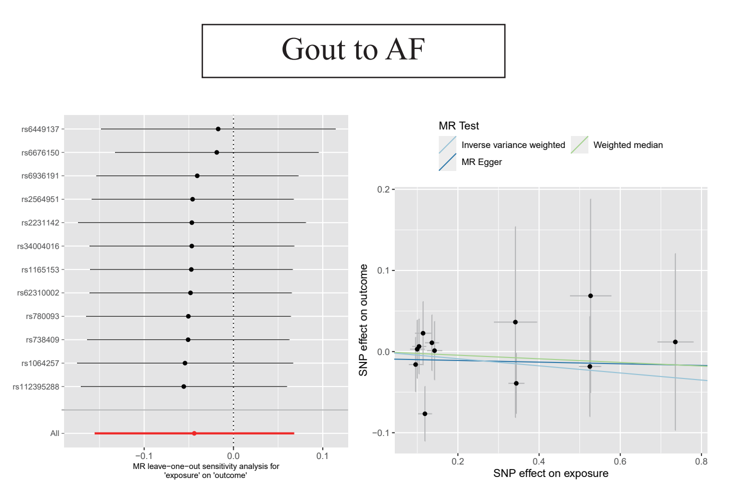

Gout to LBBB

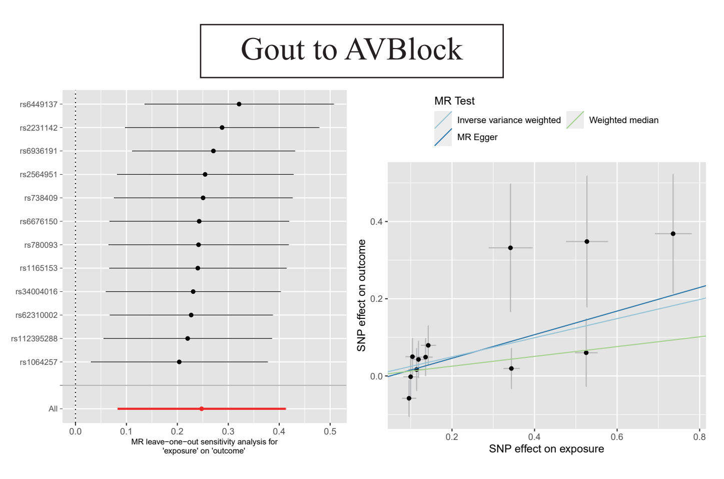

Gout to RBBB

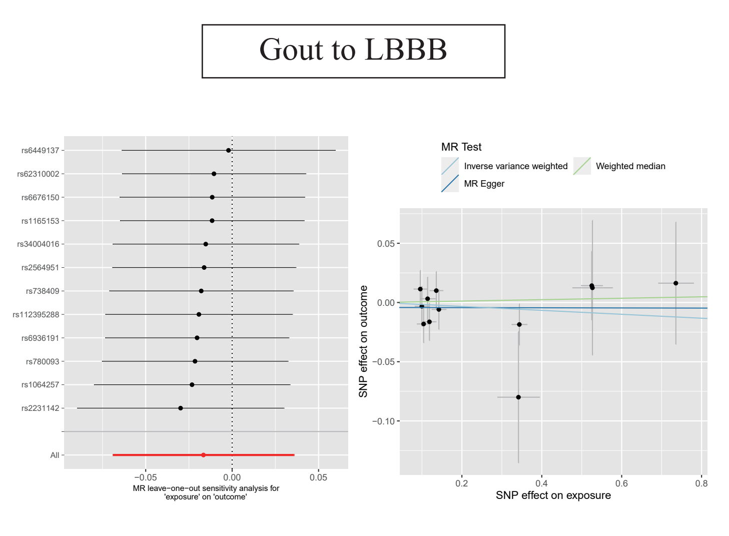

Gout to PT
