# Supplementary material for: Causal association between common rheumatic diseases and arrhythmia: a Mendelian randomization study
Source: Front Cardiovasc Med. 2024 Oct 1;11:1419466. doi: 10.3389/fcvm.2024.1419466 (PMC11473426; doi:10.3389/fcvm.2024.1419466)
Supplement: Supplementary file 2 [file Table1.docx]

***Supplementary Material***

**Genetic Causal Association between Common Rheumatic Diseases and arrhythmias: A Mendelian Randomization Study**

**Yuchen Zhang ^1^, Ke Zhang^1^, Xinai Meng^1^, Tian Liu^1^, Yanjia Chen^2^, Xingfu Huang ^1*^**

*** Correspondence:** Xingfu Huang (happyhxf@163.com)

**Supplementary Table 1**

**SNPs for the six rheumatic diseases**

Characteristics of instrumental variables for Ankylosing spondylitis

|  | **SNP** | **EA** | **OA** | **Samplesize** | **EAF** | **SE** | **β** | ***P* value** | **R²** | **F** |
| --- | --- | --- | --- | --- | --- | --- | --- | --- | --- | --- |
| 1 | rs11209026 | A | G | 10619 | 0.0616 | 0.009545 | -0.10358 | 1.94E-27 | 0.010969 | 117.7522 |
| 2 | rs41299637 | G | T | 10619 | 0.2734 | 0.00491 | -0.03905 | 1.81E-15 | 0.005922 | 63.2467 |
| 3 | rs4129267 | T | C | 10619 | 0.3598 | 0.004226 | -0.03077 | 3.32E-13 | 0.004967 | 52.99826 |
| 4 | rs1801274 | G | A | 10619 | 0.5109 | 0.004177 | 0.025318 | 1.35E-09 | 0.003448 | 36.734 |
| 5 | rs6600247 | C | T | 10619 | 0.5457 | 0.004151 | 0.032833 | 2.58E-15 | 0.005857 | 62.55532 |
| 6 | rs4672505 | G | A | 10619 | 0.6252 | 0.004151 | -0.05978 | 5.14E-47 | 0.019155 | 207.3354 |
| 7 | rs4676410 | A | G | 10619 | 0.1988 | 0.004902 | 0.028101 | 9.9E-09 | 0.003085 | 32.85496 |
| 8 | rs12615545 | C | T | 10619 | 0.4414 | 0.004173 | 0.025473 | 1.03E-09 | 0.003497 | 37.25898 |
| 9 | rs27529 | G | A | 10619 | 0.6501 | 0.004299 | -0.06204 | 3.28E-47 | 0.019236 | 208.231 |
| 10 | rs6556416 | C | A | 10619 | 0.6958 | 0.0046 | 0.025215 | 4.22E-08 | 0.002821 | 30.03929 |
| 11 | rs1041926 | A | G | 10619 | 0.9851 | 0.011692 | -0.07483 | 1.55E-10 | 0.003843 | 40.95401 |
| 12 | rs2596501 | T | C | 10619 | 0.5338 | 0.004194 | -0.15234 | 1E-200 | 0.11051 | 1319.059 |
| 14 | rs2517655 | T | C | 10619 | 0.2316 | 0.004656 | 0.088286 | 3.47E-80 | 0.032753 | 359.5163 |
| 15 | rs1128905 | C | T | 10619 | 0.4871 | 0.004095 | -0.02372 | 6.95E-09 | 0.003149 | 33.54274 |
| 16 | rs11190133 | T | C | 10619 | 0.3022 | 0.004494 | -0.03387 | 4.84E-14 | 0.00532 | 56.78141 |
| 17 | rs1250550 | A | C | 10619 | 0.2982 | 0.004305 | -0.02604 | 1.46E-09 | 0.003433 | 36.57556 |
| 18 | rs11065898 | T | C | 10619 | 0.2097 | 0.004806 | 0.026252 | 4.71E-08 | 0.002802 | 29.82734 |
| 19 | rs1860545 | A | G | 10619 | 0.4225 | 0.004354 | -0.02747 | 2.78E-10 | 0.003736 | 39.81338 |
| 20 | rs11624293 | C | T | 10619 | 0.0994 | 0.006692 | 0.042868 | 1.49E-10 | 0.00385 | 41.03188 |
| 21 | rs7191548 | C | T | 10619 | 0.335 | 0.004285 | 0.024985 | 5.51E-09 | 0.003192 | 33.99381 |
| 22 | rs9901869 | A | G | 10619 | 0.503 | 0.004089 | 0.031904 | 6.04E-15 | 0.005701 | 60.8784 |
| 23 | rs2531875 | T | G | 10619 | 0.6113 | 0.004245 | -0.02732 | 1.22E-10 | 0.003887 | 41.42904 |
| 24 | rs35164067 | A | G | 10619 | 0.168 | 0.00495 | -0.03108 | 3.43E-10 | 0.003698 | 39.40734 |
| 25 | rs2836883 | A | G | 10619 | 0.2575 | 0.004748 | -0.03968 | 6.46E-17 | 0.006533 | 69.81901 |
| 26 | rs743479 | T | C | 10619 | 0.6133 | 0.004176 | -0.02342 | 2.03E-08 | 0.002954 | 31.45867 |

Characteristics of instrumental variables for Rheumatoid arthritis

|  | **SNP** | **EA** | **OA** | **Samplesize** | **EAF** | **SE** | **β** | ***P* value** | **R²** | **F** |
| --- | --- | --- | --- | --- | --- | --- | --- | --- | --- | --- |
| 1 | rs12126142 | A | G | 58284 | 0.2969 | 0.0116 | -0.0751 | 1.013E-10 | 0.000718625 | 41.91302367 |
| 2 | rs3761959 | T | C | 58284 | 0.3069 | 0.0115 | 0.0744 | 9.64495E-11 | 0.000717611 | 41.85383785 |
| 3 | rs61828284 | T | C | 58284 | 0.0386 | 0.0348 | -0.2018 | 6.32805E-09 | 0.000576612 | 33.6255141 |
| 4 | rs2258734 | A | G | 58284 | 0.2989 | 0.0123 | -0.0921 | 6.04366E-14 | 0.000961041 | 56.06529796 |
| 5 | rs28411352 | T | C | 58284 | 0.1803 | 0.0136 | 0.0914 | 1.66418E-11 | 0.000774335 | 45.16475636 |
| 6 | rs2301888 | A | G | 58284 | 0.3847 | 0.0121 | -0.1282 | 3.75232E-26 | 0.001922296 | 112.2510486 |
| 7 | rs6679677 | A | C | 58284 | 0.0563 | 0.023 | 0.591 | 1.4093E-145 | 0.01120154 | 660.2438838 |
| 8 | rs1234313 | G | A | 58284 | 0.6075 | 0.0133 | 0.0797 | 1.89701E-09 | 0.000615739 | 35.90859873 |
| 9 | rs2076616 | G | A | 58284 | 0.6098 | 0.0135 | -0.0885 | 6.20297E-11 | 0.0007368 | 42.97383396 |
| 10 | rs10911902 | T | C | 58284 | 0.1246 | 0.0152 | -0.0847 | 2.357E-08 | 0.000532475 | 31.0502243 |
| 11 | rs28421442 | A | T | 58284 | 0.1569 | 0.0214 | -0.1234 | 7.86303E-09 | 0.000570172 | 33.24971061 |
| 12 | rs12466919 | T | C | 58284 | 0.1733 | 0.0152 | 0.1025 | 1.58891E-11 | 0.000779601 | 45.47216707 |
| 13 | rs11123811 | C | T | 58284 | 0.6512 | 0.0114 | -0.0995 | 2.01094E-18 | 0.001305329 | 76.17659492 |
| 14 | rs11889341 | T | C | 58284 | 0.2112 | 0.0129 | 0.1466 | 4.32215E-30 | 0.002210945 | 129.1438166 |
| 15 | rs1858037 | A | T | 58284 | 0.4074 | 0.0131 | -0.1012 | 1.14393E-14 | 0.00102288 | 59.67652566 |
| 16 | rs1355208 | G | A | 58284 | 0.6377 | 0.0119 | 0.0818 | 6.76862E-12 | 0.000810049 | 47.24956141 |
| 17 | rs3087243 | A | G | 58284 | 0.226 | 0.0124 | -0.1261 | 3.31513E-24 | 0.0017712 | 103.4122292 |
| 18 | rs3806624 | G | A | 58284 | 0.7654 | 0.0131 | 0.0863 | 3.93641E-11 | 0.000744057 | 43.39743858 |
| 19 | rs4602367 | G | A | 58284 | 0.6972 | 0.0117 | 0.075 | 1.75699E-10 | 0.000704523 | 41.08997721 |
| 20 | rs13103285 | T | C | 58284 | 0.4018 | 0.0131 | 0.0989 | 4.29042E-14 | 0.000976959 | 56.99478096 |
| 21 | rs34046593 | A | G | 58284 | 0.1573 | 0.017 | 0.1422 | 7.16638E-17 | 0.001199032 | 69.96590355 |
| 22 | rs403214 | G | A | 58284 | 0.5533 | 0.0146 | -0.0914 | 3.95804E-10 | 0.000671963 | 39.18968538 |
| 23 | rs2918392 | C | T | 58284 | 0.6939 | 0.0122 | 0.0668 | 4.62104E-08 | 0.000514115 | 29.97908411 |
| 24 | rs7731626 | A | G | 58284 | 0.1669 | 0.0184 | -0.1956 | 1.94089E-26 | 0.001935136 | 113.0022659 |
| 25 | rs244685 | G | T | 58284 | 0.7884 | 0.0144 | -0.089 | 6.03601E-10 | 0.00065497 | 38.19795618 |
| 26 | rs3025669 | G | C | 58284 | 0.1605 | 0.0222 | -0.2534 | 2.91273E-30 | 0.002230428 | 130.2843856 |
| 27 | rs9258357 | C | T | 58284 | 0.8345 | 0.0194 | 0.1874 | 5.76501E-22 | 0.001598423 | 93.30841457 |
| 28 | rs115521560 | C | A | 58284 | 0.5239 | 0.0364 | 0.7878 | 1.2912E-103 | 0.007972664 | 468.3971918 |
| 29 | rs9271365 | G | T | 58284 | 0.5857 | 0.0128 | 0.4888 | 1E-200 | 0.024409598 | 1458.235116 |
| 30 | rs62422878 | T | C | 58284 | 0.0879 | 0.0176 | 0.1037 | 3.57396E-09 | 0.000595284 | 34.71500836 |
| 31 | rs12530098 | T | C | 58284 | 0.1223 | 0.0204 | 0.1382 | 1.35394E-11 | 0.0007868 | 45.89240824 |
| 32 | rs1611236 | A | G | 58284 | 0.212 | 0.0131 | -0.1165 | 4.5436E-19 | 0.001355099 | 79.08504325 |
| 33 | rs71565312 | A | G | 58284 | 0.0146 | 0.0402 | 0.699 | 1.1051E-67 | 0.005160667 | 302.3342453 |
| 34 | rs212389 | A | G | 58284 | 0.4297 | 0.0147 | 0.1058 | 6.65733E-13 | 0.000887977 | 51.7990462 |
| 35 | rs112733823 | T | C | 58284 | 0.0668 | 0.0188 | 0.191 | 3.82032E-24 | 0.001767801 | 103.2134115 |
| 36 | rs139395255 | G | A | 58284 | 0.1839 | 0.0235 | 0.3833 | 8.6836E-60 | 0.004543753 | 266.0277927 |
| 37 | rs114508013 | A | G | 58284 | 0.0149 | 0.0441 | 0.488 | 1.81593E-28 | 0.002096533 | 122.4468345 |
| 38 | rs5020946 | T | G | 58284 | 0.4122 | 0.0169 | 0.6519 | 1E-200 | 0.024893796 | 1487.899678 |
| 39 | rs41316148 | C | T | 58284 | 0.4004 | 0.0876 | -0.6134 | 2.474E-12 | 0.000840552 | 49.03028616 |
| 40 | rs2233424 | T | C | 58284 | 0.1933 | 0.0187 | 0.1964 | 6.49232E-26 | 0.001888988 | 110.3023718 |
| 41 | rs7749323 | A | G | 58284 | 0.0461 | 0.0253 | 0.2835 | 3.46817E-29 | 0.002149716 | 125.5596745 |
| 42 | rs9405192 | A | G | 58284 | 0.3995 | 0.0137 | -0.089 | 9.25977E-11 | 0.000723561 | 42.20111989 |
| 43 | rs11754264 | C | T | 58284 | 0.2742 | 0.0193 | -0.1359 | 1.88105E-12 | 0.000849974 | 49.58032765 |
| 44 | rs146305655 | A | G | 58284 | 0.0141 | 0.0452 | -0.4379 | 3.29079E-22 | 0.001607773 | 93.85515209 |
| 45 | rs76153210 | T | C | 58284 | 0.2047 | 0.0205 | 0.1597 | 6.83282E-15 | 0.001040162 | 60.68581757 |
| 46 | rs1571878 | T | C | 58284 | 0.3952 | 0.0116 | -0.1539 | 4.12572E-40 | 0.003010941 | 176.0136537 |
| 47 | rs117026326 | T | C | 58284 | 0.1113 | 0.0424 | 0.381 | 2.44681E-19 | 0.001383463 | 80.74271251 |
| 48 | rs42034 | G | A | 58284 | 0.465 | 0.0153 | 0.0871 | 1.28499E-08 | 0.000555729 | 32.40697883 |
| 49 | rs740122 | A | G | 58284 | 0.4193 | 0.0134 | -0.0782 | 5.37205E-09 | 0.000583984 | 34.05563687 |
| 50 | rs4717901 | C | A | 58284 | 0.1939 | 0.0349 | 0.249 | 9.52138E-13 | 0.000872608 | 50.90177622 |
| 51 | rs3757387 | C | T | 58284 | 0.5585 | 0.0137 | 0.1236 | 1.86681E-19 | 0.00139457 | 81.39184706 |
| 52 | rs9693589 | A | G | 58284 | 0.4006 | 0.0128 | 0.1127 | 1.49589E-18 | 0.001328316 | 77.51986181 |
| 53 | rs10435844 | T | G | 58284 | 0.4942 | 0.0121 | -0.0784 | 9.72971E-11 | 0.000719779 | 41.98039125 |
| 54 | rs11574914 | A | G | 58284 | 0.1513 | 0.0149 | 0.1153 | 9.91745E-15 | 0.001026339 | 59.87853618 |
| 55 | rs3134883 | A | G | 58284 | 0.2065 | 0.0125 | 0.0991 | 1.98381E-15 | 0.001077233 | 62.85102721 |
| 56 | rs502919 | C | T | 58284 | 0.4349 | 0.0134 | 0.0829 | 6.17007E-10 | 0.000656244 | 38.27229993 |
| 57 | rs7097397 | A | G | 58284 | 0.4 | 0.012 | -0.0847 | 1.41612E-12 | 0.000854051 | 49.81835988 |
| 58 | rs71508903 | T | C | 58284 | 0.1689 | 0.0143 | 0.1487 | 3.12608E-25 | 0.001851806 | 108.1272006 |
| 59 | rs2275806 | A | G | 58284 | 0.4361 | 0.0122 | -0.0725 | 2.51397E-09 | 0.000605542 | 35.31355572 |
| 60 | rs1538981 | T | C | 58284 | 0.3568 | 0.0114 | 0.0671 | 4.41601E-09 | 0.000594057 | 34.64339413 |
| 61 | rs6479800 | C | G | 58284 | 0.4059 | 0.0181 | 0.1202 | 3.01093E-11 | 0.000756091 | 44.09982668 |
| 62 | rs7105899 | A | G | 58284 | 0.3079 | 0.0128 | -0.0797 | 4.63895E-10 | 0.00066475 | 38.76875018 |
| 63 | rs6421571 | C | T | 58284 | 0.8967 | 0.0178 | 0.134 | 5.57057E-14 | 0.0009714 | 56.67019267 |
| 64 | rs9943599 | T | C | 58284 | 0.3545 | 0.0131 | 0.083 | 2.70402E-10 | 0.00068828 | 40.14197078 |
| 65 | rs34502849 | A | G | 58284 | 0.2473 | 0.014 | -0.0851 | 1.068E-09 | 0.000633546 | 36.94776272 |
| 66 | rs4409785 | C | T | 58284 | 0.4338 | 0.017 | 0.0982 | 7.84802E-09 | 0.000572173 | 33.36646746 |
| 67 | rs660442 | A | G | 58284 | 0.0936 | 0.0175 | -0.1067 | 1.11301E-09 | 0.000637421 | 37.17387536 |
| 68 | rs4963581 | A | G | 58284 | 0.5109 | 0.0156 | 0.0856 | 3.75301E-08 | 0.000516327 | 30.10810554 |
| 69 | rs4622308 | T | C | 58284 | 0.4615 | 0.0125 | 0.0878 | 2.20699E-12 | 0.00084577 | 49.33488303 |
| 70 | rs9532434 | C | T | 58284 | 0.7956 | 0.0126 | 0.114 | 1.94089E-19 | 0.001402522 | 81.85660145 |
| 71 | rs1595260 | T | A | 58284 | 0.7851 | 0.0126 | 0.0845 | 2.28613E-11 | 0.00077106 | 44.97357637 |
| 72 | rs1950897 | T | C | 58284 | 0.5178 | 0.0144 | 0.1069 | 1.02212E-13 | 0.000944649 | 55.10811084 |
| 73 | rs2841275 | C | A | 58284 | 0.6245 | 0.0179 | 0.1617 | 1.7108E-19 | 0.00139816 | 81.60167528 |
| 74 | rs7170107 | T | C | 58284 | 0.1214 | 0.0158 | 0.1366 | 6.11223E-18 | 0.0012808 | 74.74330917 |
| 75 | rs8032939 | C | T | 58284 | 0.6126 | 0.0123 | 0.1244 | 4.46581E-24 | 0.001751942 | 102.285868 |
| 76 | rs12918327 | T | C | 58284 | 0.1118 | 0.0157 | 0.0867 | 3.04299E-08 | 0.000522953 | 30.49467345 |
| 77 | rs7206670 | T | G | 58284 | 0.3852 | 0.0119 | 0.0701 | 4.142E-09 | 0.000595024 | 34.69981906 |
| 78 | rs9927316 | G | C | 58284 | 0.2837 | 0.0136 | 0.0906 | 2.30303E-11 | 0.000760849 | 44.37758614 |
| 79 | rs4795400 | T | C | 58284 | 0.2452 | 0.012 | 0.0743 | 5.85504E-10 | 0.000657325 | 38.3354206 |
| 80 | rs2847297 | G | A | 58284 | 0.5197 | 0.0119 | 0.0903 | 2.64972E-14 | 0.000986969 | 57.57933899 |
| 81 | rs34536443 | C | G | 58284 | 0.5217 | 0.0474 | -0.3801 | 1.07895E-15 | 0.001102074 | 64.30195141 |
| 82 | rs6011186 | T | C | 58284 | 0.3342 | 0.0171 | -0.1074 | 3.19396E-10 | 0.000676353 | 39.44586091 |
| 83 | rs1883832 | C | T | 58284 | 0.7483 | 0.0127 | 0.1052 | 1.13006E-16 | 0.001175882 | 68.6134307 |
| 84 | rs7278257 | C | G | 58284 | 0.4578 | 0.0143 | -0.0894 | 4.27504E-10 | 0.000670135 | 39.08301503 |
| 85 | rs1893592 | C | A | 58284 | 0.4611 | 0.0132 | -0.0976 | 1.48115E-13 | 0.00093712 | 54.66846376 |
| 86 | rs2073609 | C | T | 58284 | 0.3599 | 0.0182 | 0.1029 | 1.46501E-08 | 0.000548151 | 31.96487943 |
| 87 | rs8126756 | C | T | 58284 | 0.5461 | 0.0137 | -0.0823 | 1.81401E-09 | 0.000618786 | 36.08640618 |
| 88 | rs2069235 | A | G | 58284 | 0.3771 | 0.014 | 0.1296 | 1.69005E-20 | 0.001468137 | 85.69175329 |
| 89 | rs5754104 | A | G | 58284 | 0.2317 | 0.0139 | 0.0891 | 1.355E-10 | 0.000704483 | 41.08761235 |
| 90 | rs5912815 | G | T | 58284 | 0.5853 | 0.0134 | -0.0787 | 4.79104E-09 | 0.000591471 | 34.4925232 |

Characteristics of instrumental variables for Systemic lupus erythematosus

|  | **SNP** | **EA** | **OA** | **Samplesize** | **EAF** | **SE** | **β** | ***P* value** | **R²** | **F** |
| --- | --- | --- | --- | --- | --- | --- | --- | --- | --- | --- |
| 1 | rs4844538 | T | A | 12653 | 0.504 | 0.0322 | -0.1883 | 4.81E-09 | 0.002695 | 34.19166 |
| 2 | rs201036579 | C | T | 12653 | 0.0129 | 0.0527 | -0.2878 | 4.85E-08 | 0.002351 | 29.81891 |
| 3 | rs146744330 | C | T | 12653 | 0.6869 | 0.0319 | 0.3457 | 2.68E-27 | 0.009196 | 117.4218 |
| 4 | rs41430444 | C | T | 12653 | 0.0905 | 0.0428 | -0.3077 | 6.57E-13 | 0.004068 | 51.67718 |
| 5 | rs11889341 | T | C | 12653 | 0.2286 | 0.0295 | 0.4164 | 3.33E-45 | 0.015502 | 199.2089 |
| 6 | rs13385731 | C | T | 12653 | 0.0577 | 0.0421 | -0.3654 | 4.19E-18 | 0.005918 | 75.31895 |
| 7 | rs7650774 | C | T | 12653 | 0.1551 | 0.0306 | -0.1798 | 4.07E-09 | 0.002721 | 34.51977 |
| 8 | rs10516487 | A | G | 12653 | 0.2972 | 0.0398 | -0.2547 | 1.64E-10 | 0.003226 | 40.94709 |
| 9 | rs244689 | G | A | 12653 | 0.841 | 0.0291 | -0.1595 | 4.29E-08 | 0.002369 | 30.0377 |
| 10 | rs10036748 | T | C | 12653 | 0.2555 | 0.0338 | 0.2021 | 2.29E-09 | 0.002818 | 35.74626 |
| 11 | rs2431697 | C | T | 12653 | 0.4314 | 0.0451 | -0.2538 | 1.84E-08 | 0.002497 | 31.66369 |
| 12 | rs16870693 | A | C | 12653 | 0.0109 | 0.0403 | 0.3849 | 1.37E-21 | 0.007158 | 91.20467 |
| 13 | rs451263 | A | G | 12653 | 0.0825 | 0.0414 | 0.3263 | 3.02E-15 | 0.004886 | 62.11047 |
| 14 | rs6941485 | G | A | 12653 | 0.2753 | 0.0314 | 0.2034 | 9.53E-11 | 0.003305 | 41.95406 |
| 15 | rs9269627 | T | C | 12653 | 0.829 | 0.0347 | -0.6737 | 9.32E-84 | 0.028929 | 376.8821 |
| 16 | rs13213165 | G | T | 12653 | 0.1869 | 0.0322 | 0.2888 | 3.13E-19 | 0.006317 | 80.42924 |
| 17 | rs3800387 | A | G | 12653 | 0.0179 | 0.0303 | 0.1731 | 1.08E-08 | 0.002573 | 32.63174 |
| 18 | rs9387400 | A | C | 12653 | 0.4145 | 0.0536 | -0.2967 | 3.14E-08 | 0.002416 | 30.63634 |
| 19 | rs16869875 | T | C | 12653 | 0.0189 | 0.04 | 0.4811 | 3.01E-33 | 0.011304 | 144.6379 |
| 20 | rs5029937 | T | G | 12653 | 0.0239 | 0.0676 | 0.6844 | 4.32E-24 | 0.008036 | 102.4844 |
| 21 | rs4134466 | G | A | 12653 | 0.5994 | 0.0306 | -0.2136 | 3.09E-12 | 0.003836 | 48.71817 |
| 22 | rs688652 | C | T | 12653 | 0.8867 | 0.0468 | -0.3154 | 1.61E-11 | 0.003577 | 45.4112 |
| 23 | rs11185603 | G | C | 12653 | 0.3539 | 0.0327 | -0.2703 | 1.34E-16 | 0.005371 | 68.31687 |
| 24 | rs76571753 | T | G | 12653 | 0.1441 | 0.0414 | 0.2792 | 1.46E-11 | 0.003582 | 45.47383 |
| 25 | rs377080180 | T | A | 12653 | 0.0785 | 0.0842 | 0.5052 | 1.99E-09 | 0.002837 | 35.99431 |
| 26 | rs1167791 | T | C | 12653 | 0.4185 | 0.0321 | 0.1981 | 7.14E-10 | 0.003001 | 38.07941 |
| 27 | rs4731532 | A | G | 12653 | 0.506 | 0.0334 | 0.3716 | 8.15E-29 | 0.009688 | 123.7627 |
| 28 | rs6993775 | T | G | 12653 | 0.8151 | 0.0346 | 0.2987 | 6.06E-18 | 0.005856 | 74.51601 |
| 29 | rs2618473 | T | C | 12653 | 0.2614 | 0.0339 | 0.359 | 2.89E-26 | 0.008785 | 112.1297 |
| 30 | rs7097397 | A | G | 12653 | 0.3956 | 0.031 | -0.2385 | 1.32E-14 | 0.004656 | 59.18133 |
| 31 | rs4930642 | G | A | 12653 | 0.9861 | 0.0344 | -0.2336 | 1.1E-11 | 0.003631 | 46.10629 |
| 32 | rs12575600 | G | C | 12653 | 0.0974 | 0.0293 | 0.276 | 4.12E-21 | 0.006964 | 88.71852 |
| 33 | rs620088 | A | G | 12653 | 0.3429 | 0.0334 | -0.2044 | 9.38E-10 | 0.002951 | 37.44555 |
| 34 | rs7486387 | A | G | 12653 | 0.1948 | 0.0314 | -0.1886 | 1.98E-09 | 0.002843 | 36.07077 |
| 35 | rs11059928 | T | A | 12653 | 0.1113 | 0.0348 | 0.2888 | 1.15E-16 | 0.005414 | 68.86003 |
| 36 | rs2841281 | T | C | 12653 | 0.4682 | 0.0292 | 0.1933 | 3.56E-11 | 0.003451 | 43.81566 |
| 37 | rs142105922 | T | A | 12653 | 0.1889 | 0.0592 | -0.4029 | 9.73E-12 | 0.003647 | 46.31082 |
| 38 | rs12599402 | C | T | 12653 | 0.4245 | 0.0292 | -0.1983 | 1.02E-11 | 0.003632 | 46.11169 |
| 39 | rs55701306 | T | C | 12653 | 0.0606 | 0.0293 | 0.1662 | 1.38E-08 | 0.002536 | 32.17052 |
| 40 | rs5749502 | A | T | 12653 | 0.1819 | 0.0289 | 0.2173 | 5.43E-14 | 0.004448 | 56.52689 |

Characteristics of instrumental variables for Sicca syndrome

|  | **SNP** | **EA** | **OA** | **Samplesize** | **EAF** | **SE** | **β** | ***P* value** | **R²** | **F** |
| --- | --- | --- | --- | --- | --- | --- | --- | --- | --- | --- |
| 1 | rs10174238 | A | G | 368028 | 0.725327 | 0.032356 | -0.2046 | 2.56E-10 | 0.000109 | 39.98367 |
| 2 | rs3117581 | G | A | 368028 | 0.175149 | 0.039758 | 0.733315 | 5.77E-76 | 0.000924 | 340.1981 |
| 3 | rs9272305 | G | C | 368028 | 0.249719 | 0.03359 | 0.420946 | 4.99E-36 | 0.000427 | 157.051 |
| 4 | rs16869677 | T | C | 368028 | 0.108818 | 0.047659 | 0.368664 | 1.03E-14 | 0.000163 | 59.83642 |
| 5 | rs113858286 | G | C | 368028 | 0.042586 | 0.075159 | 0.426326 | 1.41E-08 | 8.74E-05 | 32.17471 |
| 6 | rs150724213 | A | G | 368028 | 0.04318 | 0.075101 | 0.450504 | 1.99E-09 | 9.78E-05 | 35.9837 |
| 7 | rs2004640 | G | T | 368028 | 0.414318 | 0.028356 | -0.24807 | 2.16E-18 | 0.000208 | 76.53377 |

Characteristics of instrumental variables for Dermatomyositis

|  | **SNP** | **EA** | **OA** | **Samplesize** | **EAF** | **SE** | **β** | ***P* value** | **R²** | **F** |
| --- | --- | --- | --- | --- | --- | --- | --- | --- | --- | --- |
| 1 | rs72831235 | C | T | 365676 | 0.089264 | 0.234334 | 1.2309 | 1.5E-07 | 7.54E-05 | 27.59132 |
| 2 | rs2516482 | G | A | 365676 | 0.255245 | 0.134236 | 0.681051 | 3.91E-07 | 7.04E-05 | 25.74061 |
| 3 | rs2040406 | G | A | 365676 | 0.381119 | 0.11493 | 0.593562 | 2.41E-07 | 7.29E-05 | 26.67245 |

Characteristics of instrumental variables for Gout

|  | **SNP** | **EA** | **OA** | **Samplesize** | **EAF** | **SE** | **β** | ***P* value** | **R²** | **F** |
| --- | --- | --- | --- | --- | --- | --- | --- | --- | --- | --- |
| 1 | rs6676150 | C | G | 249351 | 0.405562 | 0.016479 | 0.118777 | 5.69E-13 | 0.000208 | 51.95104 |
| 2 | rs780093 | C | T | 249351 | 0.620732 | 0.016623 | -0.13598 | 2.83E-16 | 0.000268 | 66.91602 |
| 3 | rs2564951 | T | C | 249351 | 0.315993 | 0.017419 | 0.099806 | 1.01E-08 | 0.000132 | 32.8281 |
| 4 | rs34004016 | C | T | 249351 | 0.26868 | 0.01795 | -0.14243 | 2.11E-15 | 0.000252 | 62.96313 |
| 5 | rs6449137 | T | A | 249351 | 0.202169 | 0.019108 | -0.3439 | 2.04E-72 | 0.001297 | 323.9002 |
| 6 | rs2231142 | T | G | 249351 | 0.117451 | 0.026734 | 0.525129 | 6.67E-86 | 0.001545 | 385.8341 |
| 7 | rs62310002 | G | A | 249351 | 0.02757 | 0.052522 | 0.341661 | 7.76E-11 | 0.00017 | 42.31634 |
| 8 | rs6936191 | C | T | 249351 | 0.580735 | 0.016419 | -0.09588 | 5.23E-09 | 0.000137 | 34.10091 |
| 9 | rs1165153 | G | A | 249351 | 0.651391 | 0.016731 | 0.104239 | 4.66E-10 | 0.000156 | 38.81622 |
| 10 | rs11227299 | G | C | 249351 | 0.458322 | 0.016187 | 0.098527 | 1.15E-09 | 0.000149 | 37.04744 |
| 11 | rs1064257 | G | C | 249351 | 0.044542 | 0.04405 | 0.735633 | 1.31E-62 | 0.001117 | 278.89 |
| 12 | rs112395288 | T | C | 249351 | 0.031252 | 0.050266 | 0.526824 | 1.06E-25 | 0.00044 | 109.8442 |
| 13 | rs738409 | G | C | 249351 | 0.20624 | 0.019598 | -0.11423 | 5.59E-09 | 0.000136 | 33.9734 |
