# Supplementary material for: Causal association between common rheumatic diseases and arrhythmia: a Mendelian randomization study
Source: Front Cardiovasc Med. 2024 Oct 1;11:1419466. doi: 10.3389/fcvm.2024.1419466 (PMC11473426; doi:10.3389/fcvm.2024.1419466)
Supplement: Supplementary file 3 [file Table2.docx]

***Supplementary Material***

Causal association between common rheumatic diseases and arrhythmia: a Mendelian randomization study

**Yuchen Zhang ^1^, Ke Zhang^1^, Xinai Meng^1^, Tian Liu^1^, Yanjia Chen^2^, Xingfu Huang ^1*^**

*** Correspondence:** Xingfu Huang (happyhxf@163.com)

**Supplementary Table 2**

**Results of Cochran’s Q statistic, MR-Egger intercept test, and MR-PRESSO test**

| Variables | Heterogeneity test | | | Pleiotropy test | |
| --- | --- | --- | --- | --- | --- |
|  | Cochrane’s Q | Qdf | pval | MR-egger pval | MR PRESSO pval |
| AS to AF | 29.44 | 19.00 | 0.06 | 0.13 | 0.07 |
| AS to AVB | 32.25 | 23.00 | 0.10 | 0.37 | 0.11 |
| AS to LBBB | 26.14 | 23.00 | 0.29 | 0.15 | 0.25 |
| AS to RBBB | 29.99 | 23.00 | 0.15 | 0.72 | 0.20 |
| AS to PT | 35.64 | 23.00 | 0.04 | 0.70 | 0.07 |
|  |  |  |  |  |  |
| RA to AF | 122.21 | 83.00 | 3.32E-03 | 0.40 | 0.97 |
| RA to AVB | 109.01 | 82.00 | 0.02 | 0.32 | 0.63 |
| RA to LBBB | 72.38 | 82.00 | 0.77 | 0.36 | 0.74 |
| RA to RBBB | 92.88 | 82.00 | 0.19 | 0.73 | 0.20 |
| RA to PT | 109.67 | 82.00 | 0.02 | 0.67 | 0.89 |
|  |  |  |  |  |  |
| SLE to AF | 55.37 | 32.00 | 6.34E-03 | 0.51 | 0.07 |
| SLE to AVB | 58.59 | 33.00 | 3.96E-03 | 0.52 | 0.50 |
| SLE to LBBB | 34.27 | 33.00 | 0.41 | 0.43 | 0.37 |
| SLE to RBBB | 41.75 | 33.00 | 0.14 | 0.25 | 0.15 |
| SLE to PT | 38.37 | 33.00 | 0.24 | 0.71 | 0.28 |
|  |  |  |  |  |  |
| SS to AF | 7.82 | 5.00 | 0.17 | 0.74 | 0.38 |
| SS to AVB | 4.00 | 5.00 | 0.55 | 0.44 | 0.40 |
| SS to LBBB | 4.02 | 5.00 | 0.55 | 0.23 | 0.55 |
| SS to RBBB | 4.36 | 5.00 | 0.50 | 0.79 | 0.49 |
| SS to PT | 6.08 | 5.00 | 0.30 | 0.20 | 0.22 |
|  |  |  |  |  |  |
| DM to AF | 0.36 | 2.00 | 0.83 | 0.66 | NA |
| DM to AVB | 0.35 | 2.00 | 0.84 | 0.92 | NA |
| DM to LBBB | 0.03 | 2.00 | 0.99 | 0.97 | NA |
| DM to RBBB | 1.51 | 2.00 | 0.47 | 0.95 | NA |
| DM to PT | 2.34 | 2.00 | 0.31 | 0.71 | NA |
|  |  |  |  |  |  |
| Gout to AF | 8.66 | 11.00 | 0.65 | 0.17 | 0.70 |
| Gout to AVB | 8.11 | 11.00 | 0.70 | 0.85 | 0.81 |
| Gout to LBBB | 6.93 | 11.00 | 0.81 | 0.67 | 0.88 |
| Gout to RBBB | 12.03 | 11.00 | 0.36 | 0.63 | 0.36 |
| Gout to PT | 6.69 | 11.00 | 0.82 | 0.67 | 0.86 |
|  |  |  |  |  |  |
