# Supplementary material for: Causal association between common rheumatic diseases and arrhythmia: a Mendelian randomization study
Source: Front Cardiovasc Med. 2024 Oct 1;11:1419466. doi: 10.3389/fcvm.2024.1419466 (PMC11473426; doi:10.3389/fcvm.2024.1419466)
Supplement: Supplementary file 4 [file Table3.docx]

Supplementary Material

# Supplementary Table 3. Characteristics of the cohorts included in the MR study

| **Phenotype** | **Description** | **Consortium/Study** | **Unit** | **Sample size** |
| --- | --- | --- | --- | --- |
| **Exposure data** |  |  |  |  |
| Ankylosing spondylitis | ICD-10 codes: M45.0 | Meta-analysis | logOR | 22,647 |
| Rheumatoid arthritis | ICD-10 codes: M06.9 | Meta-analysis | logOR | 58,284 |
| Systemic lupus erythematosus | ICD-10 codes: M32.9 | Meta-analysis | logOR | 12,653 |
| Sicca syndrome | ICD-10 codes: M35.0 | FinnGen | logOR | 365,533 |
| Dermatomyositis | ICD-10 codes: M331 | FinnGen | logOR | 365,533 |
| Gout | ICD-10 codes: M10.9 | FinnGen | logOR | 368,788 |
|  |  |  |  |  |
| **Outcome data** |  |  |  |  |
| Atrial fibrillation | Paroxysmal or permanent atrial fibrillation, or atrial flutter | Meta-analysis | logOR | 1,030,836 |
| Atrioventricular block | Includes ICD-10 codes: I440-I442 (First degree, second degree and third degree atrioventricular block), I443 (Other and unspecified atrioventricular block) | FinnGen | logOR | 75,885 |
| LBBB | Includes ICD-10 codes: I444-I445 (Left anterior and Left posterior fascicular block), I446 (Other and unspecified fascicular block), I447 (Unspecified LBBB) | FinnGen | logOR | 75,392 |
| RBBB | Includes ICD-10 codes: I450 (Right fascicular block), I451 (Other and unspecified RBBB) | FinnGen | logOR | 75,369 |
| Paroxysmal tachycardia | Includes ICD-10 codes: I47 ( Re-entry ventricular, supraventricular, ventricular tachycardia and unspecified paroxysmal tachycardia) | FinnGen | logOR | 57,999 |

| **Phenotype** | **Gender** | | **Ancestry** | **IEU ID** | **Summary data URL** | **PMID** | **URL for detailed phenotype description** |
| --- | --- | --- | --- | --- | --- | --- | --- |
| **Exposure data** |  | |  |  |  |  |  |
| Ankylosing spondylitis | Males and Females | | European | ebi-a-GCST005529 | https://gwas.mrcieu.ac.uk/datasets/ebi-a-GCST005529/ | 23749187 | https://pubmed.ncbi.nlm.nih.gov/23749187/ |
| Rheumatoid arthritis | Males and Females | | European | ebi-a-GCST90013534 | https://gwas.mrcieu.ac.uk/datasets/ebi-a-GCST90013534/ | 33310728 | https://pubmed.ncbi.nlm.nih.gov/33310728/ |
| Systemic lupus erythematosus | Males and Females | | East Asian | ebi-a-GCST90011866 | https://gwas.mrcieu.ac.uk/datasets/ebi-a-GCST90011866/ | 33536424 | https://pubmed.ncbi.nlm.nih.gov/33536424/ |
| Sicca syndrome | Males and Females | | European | finn-a- M13_SJOGREN | gs://finngen-public-data-r9/summary_stats/finngen_R9_M13_SJOGREN.gza | NA | NA |
| Dermatomyositis | Males and Females | | European | finn-a- M13_DERMATOMYOTH | gs://finngen-public-data-r9/summary_stats/finngen_R9_M13_DERMATOMYOTH.gz | NA | NA |
| Gout | Males and Females | | European | finn-a-GOUT_NOS | gs://finngen-public-data-r9/summary_stats/finngen_R9_GOUT_IDIO.gz | NA | NA |
|  |  | |  |  |  |  |  |
| **Outcome data** |  | |  |  |  |  |  |
| Atrial fibrillation | Males and Females | | European | ebi-a-GCST006414 | https://gwas.mrcieu.ac.uk/datasets/ebi-a-GCST006414/ | PMID: 30061737 | https://pubmed.ncbi.nlm.nih.gov/30061737/ |
| Atrioventricular block | Males and Females | European | | finn-a-I9_AVBLOCK | https://gwas.mrcieu.ac.uk/datasets/finn-a-I9_AVBLOCK/ | PMID: 33318493 | http://r2.finngen.fi/pheno/I9_AVBLOCK |
| LBBB | Males and Females | European | | finn-a-I9_LBBB | https://gwas.mrcieu.ac.uk/datasets/finn-a-I9_LBBB/ | PMID: 33318493 | http://r2.finngen.fi/pheno/I9_LBBB |
| RBBB | Males and Females | European | | finn-a-I9_RBBB | https://gwas.mrcieu.ac.uk/datasets/finn-a-I9_RBBB/ | PMID: 33318493 | http://r2.finngen.fi/pheno/I9_RBBB |
| Paroxysmal tachycardia | Males and Females | European | | finn-a-I9_PAROXTAC | https://gwas.mrcieu.ac.uk/datasets/finn-a-I9_PAROXTAC/ | PMID: 33318493 | http://r2.finngen.fi/pheno/I9_PAROXTAC |
